# Varicocele1Delivered at the Sisters of Charity Hospital, July 10, 1898.

**Published:** 1898-12

**Authors:** P. J. Candee


### Clinical Lecture.

### VARICOCELE.[1]

By BYRON H. DAGGETT, M. D., Buffalo, N. Y.

REPORTED BY P. J. CANDEE, M. D.

VARICOCELE is a term derived from the Latin word varus, meaning "bent awry" and kala, a Greek word, meaning tumor, and it is used to designate an enlarged and tortuous condition of the veins of the pampiniform plexus. This plexus is made up of the veins of the testis and epididymis and forms the bulk of the cord. The spermatic artery is located in front of the vas and is accompanied by the spermatic nerve. The vas also has an artery closely associated with it in its course.

Varicocele occurs nine times on the left side to once on the right. The pampiniform plexus is arranged into three groups of veins. (This is an indistinct and arbitrary division.) The anterior group is much the largest and frequently surrounds the spermatic artery. This group is first affected and is the most extensively involved. The middle group follows the vas and its artery. The smaller posterior group is made up by the veins from the tail of the epididymis.

1. Delivered at the Sisters of Charity Hospital, July 10, 1898.

Varicocele is an affection of puberty and young manhood and ceases in middle life. The veins of the pampiniform plexus dilate, elongate and become tortuous and their walls are thickened by inflammatory formation of fibrous tissue, as well as muscular hypertrophy, and it has been alleged that there may be a congenital thickness of structure.

Many causes are given to account for this abnormal condition The alleged causes are as follows: (1) Congenital abnormality; (2) persistence of the veins of the Wolfian body and duct; (3) venous hypertrophy; (4) traumatism by lifting, straining, and the like; (5) chronic meso-phlebitis; (6) absence or defectiveness of valves; (7) masturbation, excessive venery as well as continence; (8) rapid development of the sexual apparatus; (9) engorged portal circulation; (10) sigmoid pressure; (11) vis a tergo through the spermatic artery.

*Symptoms.*—Relaxed scrotum, upright posture, coughing gives a slight impulse to the veins. They become empty in a horizontal position and full on pressure over the external abdominal ring.

This malady is painless in the robust and healthy, but in weak and anemic subjects it causes aching and pain in the testicles, cords, loins and frequently in the back.

The delicate young man complains of pain, tenderness and wasting of the testes; he becomes morbid, depressed and melancholy, lest virility be impaired and impotency result. Masturbators are especially prone to morbid fears of approaching impotency. This malady comes on painlessly and insidiously, and is first noticed as a swelling, with relaxation of the scrotum.

If the veins enlarge before puberty, they may interfere with the growth and development of the testicles. If they do not enlarge until after puberty, the size of the testicle is not affected, but this organ may be less firm and elastic owing to impaired nutrition and function, due to the stagnant condition of its blood supply.

Varicocele does not cause atrophy of the testicles in the adult. The treatment is palliative and operative. The palliative treatment consists of the use of a well-fitting suspensory bandage. In the vast majority of cases there is no danger of atrophy or impairment of virility and no occasion for operation. Marriage and a healthy sexual hygiene usually result in the relief of this malady.

An operation is justifiable—first, when it debars patient from entering one of the public services and whose vocation involves much activity in the upright position. If the patient be clearly hypochondriac or a monomaniac in genital matters, an operation will not relieve his mental condition. The operation of choice is an open incision. This permits the surgeon to operate with precision and to include only that portion of the cord which is safe to dispose of. An incision is made about an inch and a half long, over the veins protruded, after grasping the affected side of the scrotum, and with one or two strokes of the scalpel the sack of enlarged veins is exposed and carefully opened. The surgeon then passes a steel director, first at the upper and then at the lower angle of the wound, piercing the packet in each place and leaving about a third of the veins behind it. He then passes a needle or probe, carrying a ligature along the groove of each director, and ties these ligatures around the included veins. These ligatures will take in a section of one and a half inches of the veins. After ligation, a pair of scissors is run along the director andthe packet is cut through, about a quarter of an inch from the proximal side of each ligature. This included portion of the varicocele is then trimmed way, and any cross branches may be secured by ligature. The two stumps then are brought together, either by uniting the threads of the ligatures or by introducing a new ligature through each stump and drawing them together in that way. Care should be exercised to co-apt the stumps. One object of this operation is to shorten the cord, and to restore the natural suspension of the testicles. The wound is then carefully examined and every bleeding point is secured, a drain inserted, after which the edges of the incision are carefully adjusted, the parts dressed with antiseptic gauze and supported by a spica bandage.

Mr. Bennett in a proposed operation for varicocele does not open the sheath immediately surrounding the veins, but passes the ligature around the entire mass, and thus secures all the affected veins as none of them lie outside of the fascia. Mr. Bennett alleges that the spermatic artery is not displaced with the vas deferens, but remains with the veins, and maintains that the artery may be safely divided with the veins, for so long as the wound remains aseptic the artery to the vas deferens and some other of the outlying branches to the spermatic artery are sufficient to carry on the blood supply to the testicle and to prevent any risk of atrophy.

Jacobson claims that a division of all the veins involves a risk. He also alleges that it is not safe to trust to the artery of the vas and outlying branches of the spermatic artery. These vessels are small and delicate and inflammation about these parts may be sufficient to choke them and cause sloughing or wasting of the organ. Jacobson advises the adoption of the method described above, which includes the excision of only about two-thirds of the enlarged mass of veins, believing that the chances of recurrence of the disease is less grave than the danger of destroying the testicle, as is often the result when the Bennett operation is done by some other one than Bennett himself. Subcutaneous ligature of the vessels is a surgical procedure that has been almost entirely superseded by the open operation since aseptic surgery has come in vogue.

This young man has a medium-sized varicocele, and desires its removal so that he may enter the public service. I shall do the open or Bennett operation, at the same time heeding the precaution given by Jacobson. I have seen three young men, during the past summer, who have each lost the testicle following the operative procedure described and proposed by Bennett.

